# Prokaryotic Super Program Advisory Committee DOE Joint Genome Institute, Walnut Creek, CA, March 27, 2013

**DOI:** 10.4056/sigs.4638348

**Published:** 2013-07-30

**Authors:** George M. Garrity, Jill Banfield, Jonathan Eisen, Niels van der Lelie, Trina McMahon, Doug Rusch, Edward DeLong, Mary Ann Moran, Cameron Currie, Jed Furhman, Steve Hallam, Phil Hugenholtz, Nancy Moran, Karen Nelson, Richard Roberts, Ramunas Stepanauskas

**Affiliations:** 1 Microbiology & Molecular Genetics, Michigan State University, East Lansing, MI; 2 Earth and Planetary Science, University of California, Berkeley, Berkeley, CA; 3 Evolution and Ecology, University of California, Davis, Davis, CA; 4 Center for Agricultural and Environmental Biotechnology, RTI International, Research Triangle Park, NC; 5 Civil and Environmental Engineering, University of Wisconsin, Madison, WI; 6 Center for Genomics and Bioinformatics, Indiana University, Boomington, IN; 7 Civil and Environmental Engineering,, Massachusetts Institute of Technology, Cambridge, MA; 8 Department of Bacteriology, University of Wisconsin, Madison, WI; 9 Department of Marine Sciences, University of Georgia, Athens, GA; 10 Department of Biological Sciences, USC, Los Angeles; 11 Department of Microbiology and Immunology, University of British Columbia, Canada; 12 Centre for Ecogenomics, University of Queensland, Brisbane, Australia; 13 Microbial Diversity Institute, Yale University, New Haven, CT; 14 J.Craig Venter Institute, Rockeville, MD; 15 New England Biolabs, Ipswich, MA; 16 Bigelow Lab for Ocean Science, East Boothbay, MA

## Abstract

The Prokaryotic Super Program Advisory Committee met on March 27, 2013 for their annual review the Prokaryotic Super Program at the DOE Joint Genome Institute. As is the case with any site visit or program review, the objective is to evaluate progress in meeting organizational objectives, provide feedback to from the user-community and to assist the JGI in formulating plans for the coming year. The advisors want to commend the JGI for its central role in developing new technologies and capabilities, and for catalyzing the formation of new collaborative user communities. Highlights of the post-meeting exchanges among the advisors focused on the importance of programmatic initiatives including:

• GEBA, which serves as a phylogenetic “base-map” on which our knowledge of functional diversity can be layered.

• FEBA, which promises to provide new insights into the physiological capabilities of prokaryotes under highly standardized conditions.

• Single-cell genomics technology, which is seen to significantly enhance our ability to interpret genomic and metagenomic data and broaden the scope of the GEBA program to encompass at least a part of the microbial “dark-matter”.

• IMG, which is seen to play a central role in JGI programs and is viewed as a strategically important asset in the JGI portfolio.

On this latter point, the committee encourages the formation of a strategic relationship between IMG and the Kbase to ensure that the intelligence, deep knowledge and experience captured in the former is not lost. The committee strongly urges the DOE to continue its support for maintaining this critical resource.

## Opening remarks

In his opening remarks, Jim Bristow (Deputy Director of JGI) informed the committee that the JGI would experience its first budget cut in the coming year. *He looked to the Advisory Committee to provide guidance on which projects should be triaged and which programs should received greater support in the future.* He emphasized that whatever path would be taken, there would be long-term ramifications. The goaal of JGI goal is to continue doing big science projects, allowing some of the smaller ones to be handled by smaller centers or individual labs. JGI wants to continue delivering not just raw data, but a complete package, preferably addressing high-profile science while balancing its service function to the community. Interestingly, the most expensive step in the program has been the submission to GenBank. Other challenges to the program include getting the DNA for sequencing hundreds of microbial genomes, “managing” large numbers of users and facilitating collaborations on projects. The possibility of creating a user forum was proposed as a possible solution.

## Overview of the Prokaryotic Super Program

Nikos Kyripdes provided the committee with a status report of the current program for prokaryotes at the JGI. He placed these into two major categories: organizational changes and informatics challenges brought on by technical advances in sequencing technology. Organizational changes included two personnel changes, with Amrita Pati assuming the responsibilities of Kostas Mavromatis, following his departure from JGI and Matt Haynes joining the group to provide cross program project management support. Organizationally, the CPS has been consolidated into four thematic areas. Three of the areas are comprised of formerly separate programs (isolates, metagenomes and single cell sequencing). The fourth area is comprised of plant microbiomes, which are a new area that has experienced an increase in demand.

Continued increase in sequencing throughput and new ‘omics data has resulted in a concomitant increase in demand for informatics services, both in support of the CSP Program (i.e. JGI users) and from the broader community wanting to use IMG resources for sequence analysis and annotation. This poses a major challenge and guidance from the committee is sought.

Major informatics challenges fall into three areas: sequence annotation, data management and scale-up to accommodate the increase in both the number of genes and genomes that are identified and classified on a weekly basis. In the case of annotation, the lack of standards for gene calling poses a significant problem as different gene calling methods can yield significantly different answers. A multi-center workshop was discussed which sought solutions to the problem. The approach that was taken was for the participants to download and integrate all publically available proteomics experiments and independently re-annotate the same genomes, then make comparisons. The results of this effort point to specific guidelines in terms of optimum methods to use for gene prediction and are currently prepared for publication by the multi-center consortium.

Data management challenges have focused on the integration of the multiple independent IMG databases to a single one and required development of new functionality and incorporation of analytical tools for single cell genomes and Omics data. Integration of metagenomes into file systems also posed a challenge. Work on improved gene annotation is underway, specifically targeting unassembled Illumina data to provide support for handling 250 M to 1.3B genes/week.

Computational grand challenges for the program include development of pangenomes and protein clusters to analyze metagenomic data. In the case of pangenomes, one needs to start by grouping “genomes of the same species” using either assigned taxonomy names or 16S sequence similarity. At the time of the meeting, 500 species had been analyzed, most of which were pathogens or host-associate strains. It was learned that when ANI (average nucleotide identity) was used, a sharp drop of its value at 96.5% was observed, which roughly correlates with 97% 16S similarity. At that level, cliques of strains were apparent, some of which included more that one type strain. Significant overlap among many groups was also observed.

Defining and analyzing protein clusters/families in sequenced metagenomes is also considered a grand informatics challenge. At present, less than 30% of the protein coding genes can be clustered with available tools (such as Pfam, COGs and KO) and as a result, only this fraction of the genes can be compared across various metagenomic datasets. New methods that would allow the efficient and scalable clustering of all the novel genes identified in metagenomes are needed and are currently under development at the JGI’s Prokaryotic program

A lively discussion followed Nikos’ update, focused mainly on the approaches that could/should be used for protein clustering, how genes and protein families are called and what thresholds should be applied. This confirms the need for standards in this area, but it may still be too early for stable standards to emerge from the community. The apparent overlap between the pangenome and clustering projects was also discussed.

## Overview of the Microbial Program

Tanja Woyke opened her discussion of the Microbial Program with a broad overview of the program structure, program products, throughput, metrics and new directions. She also discussed the role of key non-JGI participants, including the DSMZ and ATCC, the Bigelow Marine Laboratory and NERSC. The direction of the program has been steadily moving from finished genomes to draft genomes, driven by a change in community demand and cost consideration. This has allowed the JGI to produce >1,000 high quality microbial draft genomes/year with a concomitant 90% decrease in cost, driven largely by changes in sequencing technology. However, this has also resulted in generation of draft genomes consisting of an increasing number of contigs. This trend will likely change in late 2013 or 2014 as the program switches over to a PacBio only approach with a 10kb library, and will yield genomes with a single contig per replicon at a cost of approximately $2k/genome.

There has been a significant increase in the demand for single-cell sequencing. This has stimulated JGI to develop more targeted approaches of selection. At present, selection has been based on 16S sequence similarity, but this will move towards a function-driven approach in the future, coupled with pre-enrichment of uncultivated microbes from the environment prior to selection. This approach eliminates the need for genetic markers. A second approach that is under investigation is RAMAN spectroscopy, which is a fast non-invasive, non-destructive method to provide a molecular signature of an organism, prior to sequencing. Two ETOP proposals were received to address current limitations of the method (weak signal and data interpretation of the rich spectrum of >1,000 RAMAN bands).

Tanja then discussed the workflow of the function-driven approach to single cell genomics and how it will fit in to the target selection process and how it might also be used in targeted metagenomics.

Currently, projects in the Microbial Program fall into two major categories: isolates and single cells, with the former comprising the bulk of the projects from both Bioenergy Research Centers (BRC) and CSP proposals. The CSP program remains quite popular and represents the major source of genome sequencing proposals. *However, because of JGI funding constraints, the Advisory Committee was asked to consider whether or not the CSP should be restructured, with perhaps a reduction in the number of small projects coming through the quarterly calls.* While the CSP remains an excellent way to carry out pilot projects or to meet the needs of smaller or specialized labs, it has a high project-management burden. This is not a problem with the annual large-scale projects, which typically target large numbers of similar isolates or single cells. Tanja then went on to discuss the Microbial Earth Project (an extension of the GEBA project), which provides a new opportunity for community involvement through the “adopt a type strain” option.

Strains also flow into the Microbial Program through JGI Grand Challenge projects, which currently include the GEBA *Cyanobacteria* and root-nodulating bacteria (RnB) projects, the Microbial Earth Project, which focuses on type strains of validly published species of *Bacteria* and *Archaea*, the Microbial Dark Matter project and *Arabidopsis* endophyte project. These projects typically generate hundreds to thousands of isolate/samples, but the DNA is provided by a small number of well-qualified providers on a scheduled basis, thereby providing an economy of scale and reducing some of the project management burden.

Future Grand Challenge projects were then discussed. High on the list is RAMAN spectroscopy (discussed above). There are three submissions to the ETOP project, one of which will ultimately be selected for reduction to practice. Discussions ensued about the working of this tool and how it can be used to identify various cellular storage products. Data challenges were also discussed, especially the complexity of RAMAN spectra and the need to build a database to make a system useable for strain identification. A second grand challenge that was discussed was deep single-cell sequencing from a single environment. Numerous candidate environments are possible.

The Pangenome challenge was then discussed, briefly. While many aspects of the project are similar to isolate sequencing projects, this project would allow one to understand various pathways without having to know the individual strain from which it was derived. This has the potential of offsetting some of the difficulties and expense of DNA sourcing and library construction.

## Overview of the Metagenome Program

Susannah Tringe’s discussion mirrored Tanja’s (by design), and provided the Advisory Committee with a global overview of the metagenome program products and metrics. The number of products was increased in 2013 with the introduction of minimal metagenomes (pooled runs). This approach significantly increases throughput and helps to meet community demand. At the time of the meeting, output was well ahead annual projections. Susannah also discussed work on expression profiling projects, which continue to present a challenge because of RNA limitations, but progress had been made at overcoming some of the technical issues.

Historically, project cycle time has been problematic. Richard Pope conducted a study to determine where in the workflow bottlenecks occurred. These occurred at points where sequence data was “handed off” to assembly, to QC, annotation, etc., and typically involved some form of manual intervention that resulted in significant delays. Some automation of the workflow was added to help remedy the problem and resulted in a significant reduction in time to completion.

Susannah noted that there were no revolutionary technical changes in the products offered by the program in the past year. Rather, changes were evolutionary. Examples of improvements included a switch to MiSeq 16S V4 tags (2 × 250bp) that included random bases in primers. Peptide nucleic acid tags were also introduced that can bind to specific targets (e.g., chloroplasts but not mitochondria) in plant microbiomes yielding significant improvements in the recovery of bacterial and archaeal sequences. Production runs using MiSeq tags have leveled off at around 2,400 samples/quarter with roughly 30,000 reads per sample. This is a significant increase in throughput over pyrotags, which was roughly 200 samples/quarter. In the area of metatranscriptomics, depletion methods using Ribozero kits has allowed the improvement in throughput using roughly 2 µg/DNA. The method is successful even in cases where non-rRNA is less than 1% total rRNA. Finally, she discussed improvements in low-input library construction using the Mondrian microfluidic system in the Ovation SP ultralow library system. Methods have been refined to the point where it is now possible to construct libraries from as little as 1ng DNA for isolates. Application to metagenomes is planned in the future. This approach can replace more difficult methods for amplifying DNA from actively growing cells from an environment, such as BrDU labeling.

In the area of technology development, only a few microbial community proposals were submitted in response to the ETOP call. Two focused on progess in assembly methods that were solicited for full proposals, because there is considerable room for improvement in that area.

At present, the user community falls into three broad categories: BRC projects (30%), CSP projects (60%) and grand challenge projects (10%). In the CSP, terrestrial carbon cycling proposals addressed questions about permafrost soils, soil warming, soil hydrogen effects and amazon deforestation. All are currently in the prep phase. Four marine proposals were also received and accepted, although the marine environment is not considered a high priority environment by the CSP as there are numerous other programs targeting this niche. A number of other “one-off” projects covering a wide range of environments were also accepted (mixed fresh water, insect gut, a biogas plant).

The grand challenge projects in the metagenome program focused on the rhizosphere of *Arabidopsis* and its impact on plant health. Phase one of the study was completed and published in Nature in August 2012. The project was done using pyrotag sequencing (100 samples × 10 replicates) and looked at a wide variety of variables and the complex relationships between the host plant, endo and ectophytes and soil type. There was strong evidence of a plant genotype effect on the community that was reproducible. A longer-term goal is to expand these approaches to other targeted plants, including maize, agave, grasses, and poplar. This would leverage existing plant genomes and provide a much better understanding of the complex relationships that exist between a plant and its natural endophytic and ectophytic bacterial species.

Future grand challenges that Susannah discussed were the concept of flagship environments and plant species, along with targeted metagenomes that could be paired with targeted single cell genomics. She also proposed that a plant microbiome project would be highly attractive in helping to shed light into the complex relationships that exist between a plant and the microbes with which it is associated.

A lively discussion followed the presentation, with the first and obvious topic of what methods are needed to better understand the relationship between plants and other eukaryotes. Limitations in current methods, especially in metatranscriptomics were viewed as problematic. Interactions between plants and prokaryotes were less problematic and the possible use of single cell methods looked especially promising as one of the future grand challenges. Functional targets were also thought to be preferable to specific niches of purely academic interest as it would allow layering of different data types in ways that could inform further experiments in the wet lab.

As for technical needs, the metagenomics program is able to leverage methodological developments from the isolate program. But, data integration from the genome program doesn’t always exist in a form that is immediately useful and accessible. Another less obvious problem is the lack of a tracking system that allows the integration of data across programs.

## Cross-JGI Projects

Matt Haynes provided the Advisory Panel with an overview of ongoing activities to integrate the workflow and output of the CSP into a more unified program. He opened the briefing by comparing and contrasting the genome and metagenome programs, with the former having a relatively clean and simple workflow and defined set of products, but generally lacking the larger context of a metagenome project that was defining a complex environment with associated interactions among community members. On the other hand, metagenomes tended to be “more confusing”, rarely assembled to any significant extent, and used a variety of methods (16S or other marker genes, total DNA sequencing and more recently single cell genome sequencing) and covered both cultivable and non-cultivable organisms. Metatranscriptomes add to the complexity. Project data and metadata are also difficult to view in an integrated fashion. Nonetheless, there are great benefits to using isolate and single cell genomes to both aid in the assembly and interpretation of metagenomes and metatranscriptomes and metagenomes to put genomes and transcriptomes into a larger biological context.

Matt described several current CSP projects that combine all elements into a single project. What these projects teach is that the workflow for isolates, single-cells, metagenomes, transcriptomes and metatranscriptomes is separate and involves two – four project managers who may not be communicating with one another and may be working in an asynchronous manner that can span several years. A prolonged discussion followed Matt’s presentation, focusing on the need for developing a more unified pipeline and improved communication, not only within the JGI, but also with the users. Discussion about delays arising on the user side (failure to send DNA in a timely manner, failure to provide metadata) was considered a major problem that needs to be addressed, perhaps with better-defined policies that establish concrete conditions/expectations as a condition of project support, or by requiring MIMs compliance as a condition of approval of sample submission. Assembly and annotation methods, especially those that involve outsider users (the broader public rather than the PI) are also issues that fall outside of the JGI mission and is in the domain of the Kbase. *How to best support both communities is the essential question and input from the Advisory Committee is needed*.

## Technologies

### FEBA – Functional Encyclopedia of *Bacteria* and *Archaea*

Jim Bristow briefed the Advisory Committee on the rationale and progress of the FEBA project. He opened by stating that the JGI mission is increasingly slanted towards functionalization of genomic information. The reason for this shift is that the sequence data only tells us *who is there* in the environment. What we ultimately want to know is *what they are doing*, and this requires improvement in functional genome annotation. While annotation of known organisms and their determination of their close relatives by homology works well, computational methods fail for the more distantly related species. What is needed are experimental tools that can improve the functional annotation of subsets of genes across phylogenetic space. The approach that is being taken is the creation of mutant libraries by transposon bombing, which are then screened under a variety of conditions.

A proof of concept (POC) study was done using *Shewanella oneidensis* as the test organism. At present the function of 36% of the genes is unknown. Mutant libraries were generated using barcoded transposons and the library was screened for growth in a “Biolog-like” array of 300 growth conditions to determine which genes were essential for growth. Fifty of the unknown genes were determined to be essential under all conditions and 23% of the genes could be given some level of functional annotation by this approach.

The second POC experiment took 50 species from the GEBA/KMG set that provide wide phylogenetic coverage and focused on 50 carbon sources for growth (study still in progress at time of meeting). Some technical issues remained, including general utility of the single transposon to cover all species across the tree. Movement from a hybridization-based assay (microarrays) to a sequenced based assay with variably bar-coded transposons is in development. The assay is robust and scalable, but the remaining problem that needs to be addressed is applicability of the current method of transposon mutagenesis across all taxa. The potential as a new product offering by JGI looks good, if all the technical issues can be worked out.

### DNA synthesis science program

Sam Deutsch updated the Advisory Panel on this newer project at the JGI. The program has been running for a approximately 2 years and provides another approach to addressing some of the more fundamental problems of providing insight into gene function in target organisms and overcoming many of the problems of expressing genes of interest in heterologous hosts. The program addresses one of the BER’s vision for JGI as becoming a resource for biosynthetic DNA fragments for downstream applications (industrial enzymes, fully functional synthetic hosts, etc.)

Current challenges include development of a strategy and pipeline for developing fragment libraries that meet specific needs and for reducing the workflow to practice. A POC study is underway in which synthesis of the first 200 genes has been completed, 65% of which are expressed. A second POC was done in collaboration with JBEI in which themostable GH1 proteins were targeted. In that study, a set of 180 phylogenetically diverse proteins were synthesized and characterized according to substrate activity, pH range, activity in ionic liquids and thermostability.

A call for CSP projects is planned. The program can currently deliver approximately 2 Mb for synthesis projects. Current synthetic products are being archived by JGI and are available to the public, without restriction on intellectual property (IP) rights. For future projects, IP rights will belong to the individuals who request the construct, but it will also depend on the complexity of the product. Single gene products will belong to the user. Ownership of more complex constructs (e.g., large biosynthetic pathways) is not clear, especially where the JGI staff are involved in the design of the product.

### Assembly technologies

Alex Copeland briefed the Advisory Committee on current R&D activities of the Quality Control Group, which is responsible not only for the quality control, but also for assembly and reporting of this phase of the sequencing program. The QC group works for three of the four project areas and strives to maximize workflow automation, wherever possible, so that as much of the work can be pushed higher-up into the workflow. In the case of microbial genomes, the assembly process integrates with the IMG in a fully automated fashion, leaving microbial genome group to pursue improvements in existing technologies. In the case of metagenomes and the fungal program, the goal of fully automatic assembly has not yet been to achieved.

On the R&D side, a major emphasis has been on improving production aligners. A move away from Galaxy-based assembly pipelines to a pipeline based on SOAPdevo2 is under investigation to accommodate larger projects. Efficiency gains were also achieved with in-house development of techniques to merge the high number of contigs typically encountered with current sequencing methods more efficiently. Alex also discussed other work to improve assembly methods using a variety of tools and techniques to improve assembly for single cells and for obligate symbionts that are often contaminated with host DNA. Variation in contig length, misassemblies, and other anomalies for these sample types results in a constant search for improvements and each new tool that comes out is given due consideration for use. Alex discussed ongoing research with the HGAP assembler from PacBio. The PacBio approach appears to have considerable advantages over existing ones, having a very low level of mismatches, a truly random pattern of sequence error (although notoriously high) and a lack of a GC bias, which makes the method highly promising.

A lengthy discussion followed the briefing about the impact of assembly methods on the overall interpretation of genome and metagenome sequence data. There are numerous genome and sample dependencies that may not always be obvious to the end user, giving the impression that assembly is as much an art form as science. The challenge is finding the methods that work with the broadest phylogenetic coverage and results that can be assessed with objective measures.

### IMG and Portals

Victor Markowitz updated the Advisory Committee on the Integrated Microbial Genomes (IMG) data management system and the various portals used to access microbial data at JGI. In his opening comments, he stressed several times that the important thing to keep in mind is that IMG is a high throughput system that integrates microbial genome and metagenome data that must often go through multiple iterations.

IMG is a gene-centric database. It has seen a steep growth in the total number of genes in the database since these data were first collected in 2006. At the end of 2012, there were records for 29.1 M genes in the IMG that were derived from the genome-sequencing program and 15.7B genes from the metagenome program. It provides services to a community of approximately 3,100 users, worldwide, of which approximately one third are JGI collaborators. While successful, growth in the user base is difficult to sustain because it is not just the increase in the amount of data that must be considered, but also all of the cross-connections that occur as a result of integrating all of the data to provide the end users with a more comprehensive view. *The issues for the Advisory Committee to address are the allocation of resources to meet community needs and defining the scope of the intended user community*. Maintenance of the underlying code base is also problematic as the IMG relies on a number of open source tools and provides user services via browsers, which are undergoing constant change.

The IMG is divided into four basic subsystems of which microbial genomes is one. Data processing begins with data submissions (originally by email but now a fully automated process). Use of standardized terminologies and concepts are critical to data acquisition, storage and analysis and standards are applied through GOLD and automation is anchored in GOLD. An automated tracking system is now in place, but defining various processes proved far more challenging than software development. Data distribution models have also changed, going from organism specific download sites to a more integrated approach with much of the data being hosted by NERSC. Overall, the IMG system offers a very good system investment by the DOE.

IMG content consists predominantly of genomes and metagenomes, which are updated regularly, with 30% of the genomes and 70% of the metagenomes originating from JGI itself. The remainder comes from the broader community. Work is ongoing to adopt/apply community accepted standards. Architecturally, the IMG has migrated from a collection of distinct project centric systems to a unified system with a suite of domain specific interfaces, backed by a single database. IMG has also migrated from a system built on a relational database, which did not scale well, to a re-engineered system building using FastBit. Workspaces have also been built into IMG to allow users to save gene sets and function sets with computations offloaded onto a supercomputer that informs users when analyses are complete.

Although the IMG has evolved into a very useful resource that has kept pace with both user needs/expectations and developments in the field, it has not been without some cost. *How to maintain the resource going forward, and in which form, will require making some very hard decisions*.

A lively discussion followed Victor’s presentation, in response to his request for guidance. Questions he raised include how to balance the expectations of different user communities (JGI/non-JGI) and whether or not parts of the current IMG function would be better served under Kbase. Other questions involved which data should be represented in IMG (i.e., reference public genomes and metagenomes) and how all of the ‘omics data should be integrated. Should IMG be focusing on comparative analysis (expensive) or data distribution (inexpensive), or expert review/curation?

A second line of discussion focused on the IMG – NCBI relationship and whether or not IMG could serve as a repository for JGI data. What would the cost be and (tangible and non-tangible) would that fit within the JGI/DOE mission? Would the problem of perceived bottlenecks at NCBI, including delays in release of GenBank genome submissions be solved by such an approach? Could the IMG serve as an alternative host for genome information? Would the IMG eventually suffer from all of the same problems as NCBI? Could the IMG and other major databases stop submitting to GenBank and what would be the consequence?

## Strategic planning discussion

A selection of questions that had been circulated to advisory committee members were discussed during the meeting.

### Question 1.

Suggestions for additional new technologies you would consider important for the programs, in line with JGI’s future vision of moving from sequence to function. This topic was not followed up on as the discussions during the day covered many of the ongoing projects at JGI.

### Question 2. Community sequencing program

There was a consensus among the advisors that the small scale community sequencing program should be continued as it provides a great service to the broad community, but could be done at less frequent intervals than quarterly (two or three times/year). The importance of the annotation service and indexing of sequences and metadata in IMG was considered as an important aspect of the CSP.

The second part of the question was whether or not the program should remain open (unconstrained) or if a more focused approach should be applied. The committee response was slightly ambivalent, favoring some constraints to ensure that projects fit well into the overall BER mission.

### Question 3. Future grand challenge projects

1. *Continue the Phylogenetic coverage of isolates and SAGs?* - The current CSP call was focused more on functional diversity and single cell genomes as opposed to phylogenetic coverage; however, the committee members thought that the best approach might be to couple the two more closely. The broad coverage has helped in the recovery of complete genomes from metagenomes and will help to further annotation efforts. The value of sequencing type strains was also discussed, recognizing that 16S rRNA cannot adequately define a species and is at best, a weak measure of phylogenetic diversity. As such, the type strains play a major role in mapping out the phylogenetic space. Single-cell genomics is expected to play an additional “enhancing” role, serving as a bridge between phylogenetic coverage and biological process coverage.

2. *Plant Microbiome Project* - Generally thought to be a good idea, but the analysis should move from the typical within-host comparison to across-host comparisons. One suggestion was to look at quantitative trait mapping, to gain more information about the impact of the microbiome on the host.

3. *Targeted single cell genomics and metagenomics*. There was strong support from the advisory panel for the JGI to continue work in these areas. The consensus was that these approaches would have a important role in future functional genomic research. Ed Delong indicated the importance of knowing which organisms were present to interpret metatranscriptomes.

4. *Deep single cell and metagenome sequencing from one “flagship” environment* - The key will be to pick the “right” flagship. The question is whether a single environment should be selected or would that be too narrow. Also discussed was the importance of such a project to test fundamental concepts of complete integration of all of the techniques and data into a single or small number of well-defined discrete projects to demonstrate the value of applying a systems-biology approach.

5. *Large scale pangenome sequencing* - The creation of libraries is still expensive. Ideally, one would like to have 1000’s – 10,000’s of strains that could be pooled and sequenced, but the challenge would be teasing out the strain-associated data/metadata to be able to interpret the results. The possibility of receiving the DNA from a large collection of actinobacterial strains was discussed as a “low-cost” means of testing some of these concepts.

Questions arising from the Advisory Committee included one from Jill dealing with targeting phage and plasmids. Ed reiterated the point about down-stream interpretation, which could only be as good as the available metadata and picking the right pangenome to sequence would depend on knowing where the strains originated and their ecologies.

Despite the active discussion, the committee members participating in the discussion did not arrive at a decision

6. *Synthetic pangenome project* - The discussion began with the concept of creating a synthetic pangenome that might have some practical value for studying resistance to a particular disease. It quickly broadened to a more generalized discussion about the potential value and utility of the DNA synthesis capacity that was being incorporated into the CSP. What advantage might this methodology provide CSP users, besides screening for enzymes? Would these synthetic pangenomes provide a means of doing combinatorial biosynthesis? What other supporting technologies would be required (e.g., competitive assays). Would such an approach lead to an understanding of the maximum genome (as opposed to the minimum genome)?

Despite the active conversation, committee members who participated made no decision about this topic.

### Question 4. Expansion of functional genomics projects

This was considered an important direction for JGI to go, as it allows layering additional information onto the genome sequences. The next step will be to meet the expectations of the user community. The starting point will be transposon bombing, possibly with as many as 100 organisms/year. Would such a product be useful? Would there be other interaction assays that JGI should pursue?

The consensus was that this would be a valuable addition to the JGI product portfolio. However, there is a need to balance this with other approaches. Selection of projects would require careful determination about the capabilities of the end users to utilize the data and, in some cases, the capability of providing JGI with appropriate information about growth of the targeted organisms. It was unclear whether it would be possible to establish a set of “standard methods” to grow each organism under the same set of physical and biological conditions.

### Question 5. Informatics challenges

The topic of metadata availability and quality was highlighted throughout the meeting, although it was not one of the defined informatics challenges. The availability of high-quality metadata significantly enhances the value of any of the JGI products. Tools need to be available to allow the easy capture of metadata at the point where investigators have the highest interest in a given organism, typically pre-publication/pre-submission.

1.Should we *continue supporting the annotation and integration of external (i.e. non-JGI) projects in IMG?* - There was a general consensus that, under the current funding climate, JGI should focus on JGI projects. Kbase is well positioned to provide some of the services to the broader community that IMG has served in the past, including downloads

There are broader informatics challenges that IMG has historically provided and the JGI is encouraged to continue providing the necessary informatics support as it is mission critical, not only for the JGI but for the community in general. Balancing the program and community expectations needs to be carefully considered and budgeting for maintenance and support is essential. Likewise, end users need to be encouraged to provide all of the relevant metadata in a timely manner so as not to impede the JGI workflow. This is especially true with regard to metagenomic studies.

2. *Support downloading of all data (i.e. JGI and non-JGI) from IMG/portals?* - How important is submission of the isolate genomes and single cells to Genbank?

The topic was discussed and there was a general feeling among the Advisory Committee members that Genbank submission had become a major project cost and bottleneck. If an alternative approach existed that would guarantee availability of data in a consistent manner, it might prove a better long-term option, especially if it were a distributed solution.

*3. Importance of protein families from metagenomic data –* No further discussion ensued.

*4. Suggestions for additional or new ways of interacting with Kbase –* No further discussion ensued.

*5. Should we increase our offerings of “customized” analysis products?* - No further discussion ensued.

## Advisory committee membership

### Advisory committee attendees:

Jill Banfield University of California, Berkeley jbanfield@berkeley.edu
Jonathan Eisen University of California, Davis jaeisen@ucdavis.edu
George Garrity Michigan State University Garrity@msu.edu
Niels van der Lelie RTI vdlelied@rti.org
Trina McMahon University of Wisconsin tmcmahon@cae.wisc.edu
Doug Rusch JCVI drusch@jcvi.org

### Advisory committee members attending by conference call:

Edward DeLong MIT delong@mit.edu
Mary Ann Moran University of Georgia mmoran@uga.edu

### Advisory committee members not attending:

Cameron Currie University of Wisconsin currie@bact.wisc.edu
Jed Furhman USC, Los Angeles furhman@usc.edu
Steve Hallam University of British Columbia shallam@interchange.ubc.ca
Phil Hugenholtz University of Queensland phugenholta@gmail.com
Nancy Moran Yale University nancy.moran@yale.edu
Karen Nelson JCVI kenelson@jcvi.org
Richard Roberts New England Biolabs roberts@neb.com
Ramunas Stepanauskas Bigelow Lab for Ocean Science rstepanauskas@bigelow.org

